# Early-Life Exposure to Bisphenol A Damaged Pancreas That May Increase Offspring Sensitivity to High-Fat Diets

**DOI:** 10.1155/jt/6189790

**Published:** 2025-08-13

**Authors:** Fubin Chen, Huihong Zhang, Haiyan Huang, Jianjun Liu, Wei Zhu, Lingeng Lu, Yirong Xie, Hongya Li, Shurong Pi, Jingyi Zhong, Shuren Ding, Ke Zhang, Fan Wu, Bo Zhang, Yun He

**Affiliations:** ^1^Department of Toxicology, School of Public Health, Sun Yat-sen University, Guangzhou 510080, Guangdong, China; ^2^Food Safety and Health Research Center, Guangdong-Hongkong-Macao Joint Laboratory for Contaminants Exposure and Health, School of Public Health, Southern Medical University, Guangzhou 510515, Guangdong, China; ^3^Shenzhen Key Laboratory of Modern Toxicology, Shenzhen Medical Key Discipline of Health Toxicology, Shenzhen Center for Disease Control and Prevention, Shenzhen 518055, Guangdong, China; ^4^Guangzhou Center for Disease Control and Prevention, Guangzhou 510440, Guangdong, China; ^5^Department of Chronic Disease Epidemiology, Yale School of Public Health, Yale University, New Haven 06510, Connecticut, USA

**Keywords:** bisphenol A, early-life exposure, high-fat diet, lipidomics, pancreatic damage

## Abstract

Previous studies have identified early life as a sensitive window for BPA exposure that may increase the risk of metabolic disease in adulthood. However, less attention has been paid to the effects of early-life BPA exposure on the pancreas and its relationship to the development of metabolic diseases. In this study, we exposed females to 50 μg/kg/d BPA in drinking water from 6 days of gestation to weaning of offspring mice and administered a high-fat diet after weaning of offspring mice. We found that early-life BPA-exposed male mice gained body weight, had downregulated pancreatic *Ins1*, *Pdx1*, and *NeuroG3* gene expression, reduced β-cell mass, and resulted in abnormalities in glucose tolerance and insulin tolerance, whereas no significant alterations were observed in females. Lipidomic analyses of mouse pancreas using high-resolution mass spectrometry showed that early-life BPA exposure significantly altered the pancreas of offspring males. Lipid profiles of mouse pancreatic ceramidase gene mRNA expression were upregulated, enzyme activity was enhanced, and pancreatic ceramides, especially long-chain ceramides, were increased in abundance, the latter of which was closely correlated with the increased pancreatic MDA content as well as the decreased SOD enzyme activity. Taken together, our results suggest that early-life BPA exposure may increase the susceptibility of mice to a high-fat diet by altering pancreatic lipid metabolism in mice and that there are significant sex differences in this effect.

## 1. Introduction

According to the World Health Organization, there were an estimated 462 million adults with diabetes globally in 2019, and it is expected to rise to about 702 million by 2045 [1, 2]. The pathogenesis of diabetes is complex and involves various known risk factors, including genetic factors, obesity or overweight, physical inactivity, and early malnutrition [3]. However, besides these common factors, there is another factor that we may neglect, namely environmental endocrine disruptors (EDCs).

BPA is a widely recognized EDC with reproductive, developmental, and systemic toxicity, as well as obesity-inducing and weak estrogenic effects [4–6]. Many population-based studies have found a significantly positive association between the incidence of diabetes and the levels of BPA in blood and urine [7–9]. BPA has a short half-life of only 4–6 h in the body [10], but previous studies have confirmed the presence of BPA in the urine and blood across the world [7–9]. For example, more than 90% of Americans had measurable BPA (0.4 μg/L–149 μg/L) in their urine [11]. A 6-year follow-up biomonitoring study in Germany showed that BPA was detectable in all urine samples (mothers, 1.5–2.0 μg/L; children, 2.0–2.7 μg/L) [12]. After entering the body, BPA undergoes metabolization by uridine diphosphate glucuronosyltransferase (UGT) in the intestine and liver into nonobvious biological activity BPA glucuronide (BPAG) conjugate [13]. Therefore, BPA is of low toxicity for healthy adults. However, due to the low activity of UGTs in pregnant women and their fetuses, the metabolic capacity of BPA is reduced, resulting in an increased proportion of biologically active BPA, and the potential harm of early-life exposure to BPA is greater [14, 15]. Studies have analyzed BPA in breast milk, placenta, amniotic fluid, and fetal cord blood, which indicates that BPA may affect the fetus through the placenta and breast milk. Fetus development is a critical window for the organ development and also a sensitive exposure window for exogenous compounds [16]. Previous studies have demonstrated that early-life exposure to BPA impairs the development of the pancreas, especially the islets, and subsequently disrupts glucose homeostasis in adulthood [17]. However, the underlying mechanisms are still unclear.

Poor dietary habits, especially an HFD, are important causes of metabolic diseases such as diabetes [18]. HFD is widely used to establish experimental animal models of insulin resistance and metabolic syndrome, which induce a state of “lipotoxicity” characterized by insulin resistance and impaired glucose and lipid metabolism, leading to chronic metabolic diseases such as diabetes, atherosclerosis, hypertension, and coronary heart disease [19–21]. HFD affects multiple metabolic organs in the body, of which the impact of HFD on the pancreas is particularly noteworthy.

According to the developmental origins of health and disease (DOHaD) hypothesis, early-life exposure to adverse factors determines the susceptibility to chronic diseases (such as diabetes, obesity, and cardiovascular diseases) in later life [22]. In view of the ubiquitous presence of BPA, continuous and long-term exposure to BPA is difficult to avoid for pregnant women and their babies. In addition, with the continuous development of social economy and the improvement of people's living standards, nutritional excess represented by HFD is widespread [23]. Taken together, we hypothesized that early-life BPA exposure combined with postweaning HFD had adverse effects on the body's glucose and lipid metabolism. Thus, the purpose of this study was to investigate the effects of early-life BPA exposure on pancreatic islet development and its potential interaction with HFD in pancreatic lipid profiles.

## 2. Method

### 2.1. Animal Husbandry and Experimental Design

One hundred and fifty specific pathogen-free (SPF) C57BL/6J mice (50 males, 100 females; 6–7 weeks old; body weight 15–17 g) were purchased from the Guangdong Medical Experimental Animal Center (SCXK [Yue] 2018-0002, No. 44007200091510). Animal experiments were approved by the Institutional Animal Care and Use Committee of Guangzhou Center for Disease Control and Prevention. The mice were housed in barrier facilities with a relative humidity of 50%–75%, room temperature of 20°C–24°C (22 ± 2°C), and a normal light cycle (12 h light:12 h dark). Mice had free access to food and water. After 1 week of acclimation, the mice were prepared for pregnancy. By continuous mating, the mice in each group were mated at a ratio of male to female of 2:1. On the next day, the vaginal plug test was used to determine whether the female mice were pregnant, and the day of vaginal plug detection was used to determine the 0th day of pregnancy (Gestation day 0, GD0). The results of pregnancy and delivery of dams are shown in Table S1.

The experimental design and grouping are shown in Table 1. In summary, according to the NOAEL of BPA (5000 μg/kg/day) published by FDA and previous research results, 50 μg/kg/day was selected as the BPA exposure dose. Drinking water exposure was used and BPA was dissolved in ethanol (1% final concentration) to the appropriate concentration. The dams were separated randomly into two: control (ethanol drinking water was prepared in 1% of final volume) and BPA (50 μg/kg/day) groups which begin on gestation day 6 (GD6) to postnatal day 21 (PND21). To ensure the BPA content of the drinking water, the water was changed every 3 days and the amount of water consumed was measured. Food and water intakes of the dams are shown in Table S2. After weaning, the mice were fed either a normal diet (ND) or an HFD, and two mice of each sex were randomly selected from each litter. The mice were divided into two dietary groups: ND group (D12450J) and high-fat diet group (D12492). Four experimental groups were formed by combining maternal BPA exposure and postweaning dietary conditions: ND control group (Con + ND), BPA ND group (BPA + ND), high-fat diet control group (Con + HFD), and BPA high-fat diet group (BPA + HFD). Glucose and insulin tolerance tests were performed on postnatal day 91 (PND91). F1 mice were sacrificed on PND21 and PND91, respectively, and blood and pancreatic samples were collected.

### 2.2. Glucose and Insulin Tolerance Test

#### 2.2.1. Glucose Tolerance Test

After fasting for 16 h, F1 mice were tested for glucose tolerance. Three mice from each group were randomly selected (each mouse was only tested once to avoid excessive injury). The mice were first weighed, and their individual mouse number and weight were recorded. The mice were placed in a mouse restrainer, and the tail was disinfected with 75% alcohol. The tail tips were cut off by 1–2 mm, and the first drop of blood was discarded. The tail was gently stroked from the root to the tip, and a blood glucose meter test strip was used to record the blood glucose level at 0 min by touching the blood at the tail tip. After disinfection with alcohol, the mice were returned to their cages and allowed to rest for 30 min. Then, glucose solution was injected intraperitoneally at a dose of 2.0 g/kg body weight. Blood glucose levels were measured at 15 min, 30 min, 60 min, and 120 min after injection. After the test, the mice were returned to their cages and allowed to resume normal feeding.

#### 2.2.2. Insulin Tolerance Test

Three mice from each group were randomly selected for insulin tolerance testing. The mice were fasted for 4 h before the test. Similar to glucose tolerance test, the mice were experimentally prepared for insulin tolerance test. Instead, an insulin solution was injected intraperitoneally at a dose of 0.75 U/kg body weight. Blood glucose levels were measured at 15 min, 30 min, 60 min, and 120 min after injection. After the test, the mice were returned to their cages and allowed to resume normal feeding.

### 2.3. Detection of BPA in Plasma and Pancreas

#### 2.3.1. Sample Pretreatment

Fifty milligrams of pancreas was weighed, added with 1 mL of chromatographic grade methanol and BPA internal standard (final concentration of 20 ng/mL), homogenized, and sonicated for 30 min. After vortex mixing, the supernatant was centrifuged, and the supernatant was concentrated by nitrogen blowing, 100-μL chromatographic grade.

#### 2.3.2. Chromatographic Conditions

Chromatographic column: Supersil 0DS2 (2.1 mm × 100 mm, 1.8 μm); mobile phase: A (water), B (methanol); gradient elution procedure: 0∼0.5 min, 60% B; 0.5∼3.5 min, 0∼100% B, keep 2.5 min; 6∼6.5 min, 100∼20% B; hold for 2 min. Column temperature: 40°C; sample chamber temperature: 15°C; injection volume: 5 μL.

#### 2.3.3. Mass Spectrometry Conditions

Electrospray negative ion mode (ESI−); capillary voltage: −4.5 KV; ion source temperature: 150°C; desolvation; gas temperature: 500°C; desolvation gas flow rate: 800 L/h; cone hole blowback gas flow: 50 L/h; collision gas flow: 0.15 mL/min. Multiresponse monitoring (MRM) parameters are shown in Table 2.

### 2.4. Determination of Plasma Insulin

The mouse plasma was thawed on ice, and the standard substance, biotinylated antibody working solution, enzyme conjugate working solution, and working washing solution were configured according to the manufacturer's instructions. The OD values of the standard substance and the sample to be tested were determined according to the detection steps of the instructions. Finally, the standard curve was established with the concentration as the abscissa and the OD value as the ordinate to calculate the insulin content in the sample.

### 2.5. RNA Extraction and qPCR

Total RNA from the pancreas was extracted according to the manufacturer's instructions using TRIzol reagent (Thermo Scientific™, California, USA). One microgram (1 μg) of RNA was reverse transcribed into cDNA using a reverse transcription kit (TaKaRa, Kyoto, Japan). The cDNA was then amplified using SYBR® Green Real-time PCR Master Mix (Toyobo, Osaka, Japan). The thermal cycling conditions were as follows: 95°C for 30 s, followed by 40 cycles of 95°C for 5 s, 60°C for 10 s, and 72°C for 15 s. Melting curve analysis was performed to verify the specificity of qPCR. Beta (β)-actin primers specific to mouse actin were used as a control to normalize the cDNA loading. The levels of mRNA were determined by comparing the Ct values between the target gene and the internal control gene. The primers used for qPCR are listed in Supporting Table S3.

### 2.6. Immunofluorescence Analysis of Insulin and Glucagon in Pancreas

The pancreas of treated mice was fixed overnight in 4% paraformaldehyde (PFA) followed by dehydration. It was embedded in optimal cutting temperature (OCT) compound and sectioned using a cryostat to obtain 10-μm pancreatic frozen sections. The prepared pancreatic frozen sections were warmed up, washed with phosphate-buffered saline (PBS), and blocked with bovine serum albumin (BSA) for 30 min. After PBS rinsing, the sections were incubated overnight at 4°C with insulin antibody (1:500, Huabio Cat# ET1601-12, RRID:AB_3069594,China) and glucagon antibody (1:500, Bioss Cat# bsm-0933M, RRID:AB_10857678, China). The sections were then washed with PBS and incubated with fluorescent secondary antibodies (1:500, Thermo Scientific™, USA) at room temperature for 2 h in the dark. After PBS rinsing, the sections were incubated with 4′,6-diamidino-2-phenylindole (DAPI)-containing antifade mounting medium for 10 min at room temperature in the dark. Finally, fluorescence detection and image capture were performed using a laser scanning confocal microscope (Leica, Deu). The calculation of alpha and beta cell mass is shown below:

(1) $$\begin{array}{c} \text{Alpha Cell Weight }\left(\text{mg}\right)=\frac{\text{Glu }A\;\left(\text{pixels}\right)}{\text{Glu }P\;\left(\text{pixels}\right)}\times \text{Pancreatic Weight }\left(\text{mg}\right), \end{array}$$

(2) $$\begin{array}{c} \text{Beta Cell Weight }\left(\text{mg}\right)=\frac{\text{Ins }A\;\left(\text{pixels}\right)}{\text{Ins }P\;\left(\text{pixels}\right)}\times \text{Pancreatic Weight }\left(\text{mg}\right). \end{array}$$

where Glu A (pixels): The total area of the glucagon staining (pixels). Glu P (pixels): The total area of the pancreas (pixels) on the same image as the glucagon staining. Ins A (pixels): The total area of the insulin staining (pixels). Ins P (pixels): The total area of the pancreas (pixels) on the same image as the insulin staining.

### 2.7. Pancreatic Lipid Extraction and Lipidomics Analysis

The extraction of lipids followed the method as described by Matyash et al. [24]. Briefly, the pancreatic tissue (25–30 mg) was thawed on ice, and 450 μL of 0.1% ammonium acetate solution was added. After homogenization, the mixture was transferred to a 15-mL centrifuge tube, and 1.5 mL of methanol was added, followed by oscillation and mixing. Then, 5 mL of methyl-tert-butyl ether (MTBE) was added, and the mixture was incubated at room temperature for 1 h. After adding 1.25 mL of pure water and incubating at room temperature for 10 min, the mixture was centrifuged at 1000 × *g* for 10 min at 4°C. The supernatant was collected, and 2 mL of extraction solution (MTBE:CH_3_OH:ddH_2_O = 10:3:2.5) was added to the lower layer solution. The mixture was centrifuged at 1000 × *g* for 10 min at 4°C. The supernatants were evaporated using a vacuum centrifuge and then reconstituted with 100 μL of reconstitution solution (chloroform:methanol:ddH_2_O = 60:30:2.5). From each sample, 15 μL was taken to create a pooled quality control sample, while the remaining sample was transferred to an injection vial for lipid analysis.

Using an ultrahigh-performance liquid chromatography (UHPLC) system (Thermo Scientific™, California, USA), 2 μL of each sample was injected onto a reversed-phase C30 chromatographic column (150 ∗ 2.1 mm, 3 uM, Thermo Scientific™, California, USA). Mobile phase A consisted of acetonitrile-water (6:4, v/v) with 2 mM ammonium formate, while mobile phase B consisted of acetonitrile-isopropanol (1:9, v/v) with 2 mM ammonium formate. The initial mobile phase composition was 30% solvent B, with a flow rate of 0.260 μL/min. From 0 to 2 min, the mobile phase B increased linearly from 30% to 43%; from 2 to 2.1 min, it increased linearly from 43% to 55%; from 2.1 to 12.0 min, it increased linearly from 55% to 65%; from 12.0 to 18.0 min, it increased linearly from 65% to 85%; from 18.0 to 20.0 min, it increased linearly from 85% to 100% and was maintained for 5 min; finally, from 25.0 to 25.1 min, it returned to 30% and equilibrated for 3 min. To minimize instrumental error, all samples were injected randomly. After separation by the UHPLC system, gas-based mass spectrometry analysis was performed using a QExactive Plus (Thermo Scientific™, California, USA). The parameters for positive electrospray mode (ESI+) were: sprayer temperature of 350°C, spray voltage of 3.5 kV (3.5 KV), sheath gas flow rate of 40 arbitrary units (arb), auxiliary gas flow rate of 10 arb, capillary temperature of 320°C, and scanning range of 200–1800 *m*/*z*; the parameters for negative electrospray mode (ESI−) were: sprayer temperature of 350°C, spray voltage of 3.2 kV, sheath gas flow rate of 40 arb, auxiliary gas flow rate of 10 arb, capillary temperature of 320°C, and scanning range of 200–1800 *m*/*z*. Quality control samples were tested first to stabilize the instrument before sample testing. Each sample was tested three times, and a quality control sample was tested after every nine injections to evaluate the stability of the testing.

The raw data were processed using LipidSearch software version 4.1 (Thermo Scientific™, California, USA) for peak alignment, retention time correction, peak area extraction, and analytical method validation. Changes in lipid composition were analyzed at three levels: lipid group, lipid subgroup, and lipid ion. The intensity data of each lipid molecule in all samples were normalized and subjected to principal component analysis (PCA) using R 4.3.1.

### 2.8. Ceramide Synthase Activity Assay

Homogenize pancreatic tissue (50–60 mg) in PBS buffer to prepare a suspension, and then centrifuge (4°C, 4000*g*, 20 min), collect the clarified supernatant, and dilute it to measure the enzyme activities of Cers2, Cers4, Cers5, and Cers6. All samples were diluted and analyzed using the corresponding ELISA kits (Jiangsu Meimian Industrial Co. Ltd., Jiangsu, China) according to the manufacturer's instructions.

### 2.9. Detection of Pancreatic MDA Content and SOD Enzyme Activity

Pancreatic tissue (25–30 mg) were taken, and the total protein concentration in each pancreatic tissue was determined according to the manufacturer's instructions using a bicinchoninic acid (BCA) protein quantitative kit (Thermo Scientific™, California, USA). The malondialdehyde (MDA) content and superoxide dismutase (SOD) enzyme activity in the pancreatic samples were detected using a lipid peroxidation (MDA) detection kit (Beyotime, Shanghai, China) and a total SOD activity detection kit (Beyotime, Shanghai, China), respectively. The protein concentration was used for normalization.

### 2.10. Statistical Analysis

Statistical analysis was performed by SPSS 25.0, and Shapiro–Wilk test was used to test whether the measurement data conformed to the normal distribution. Normal distribution data are expressed as mean ± standard deviation. One-way analysis of variance or two-way analysis of variance was used for significance test, and Dunnett's *t* test was used for post hoc comparison. Pearson correlation analysis was used for correlation analysis, and the test level was *α* = 0.05. The results of this study showed no difference between male and female, so the experimental data of male and female mice were combined and counted.

## 3. Result

### 3.1. General Situation of BPA Exposure in Early Life Combined With HFD Mice After Weaning

The concentrations of free BPA in the plasma and liver of maternal mice, as well as in the placenta and liver of offspring mice on postnatal day 1 (PND1), were quantified, with results presented in Table 3. The concentration range of free BPA in the plasma and liver of the BPA-exposed maternal group, as well as in the placenta and liver of PND1 offspring, was established at 4.14–33.01 ng/mL. However, free BPA was undetectable in the pancreas of offspring mice on PND21 or PND91.

**Figure 1:**
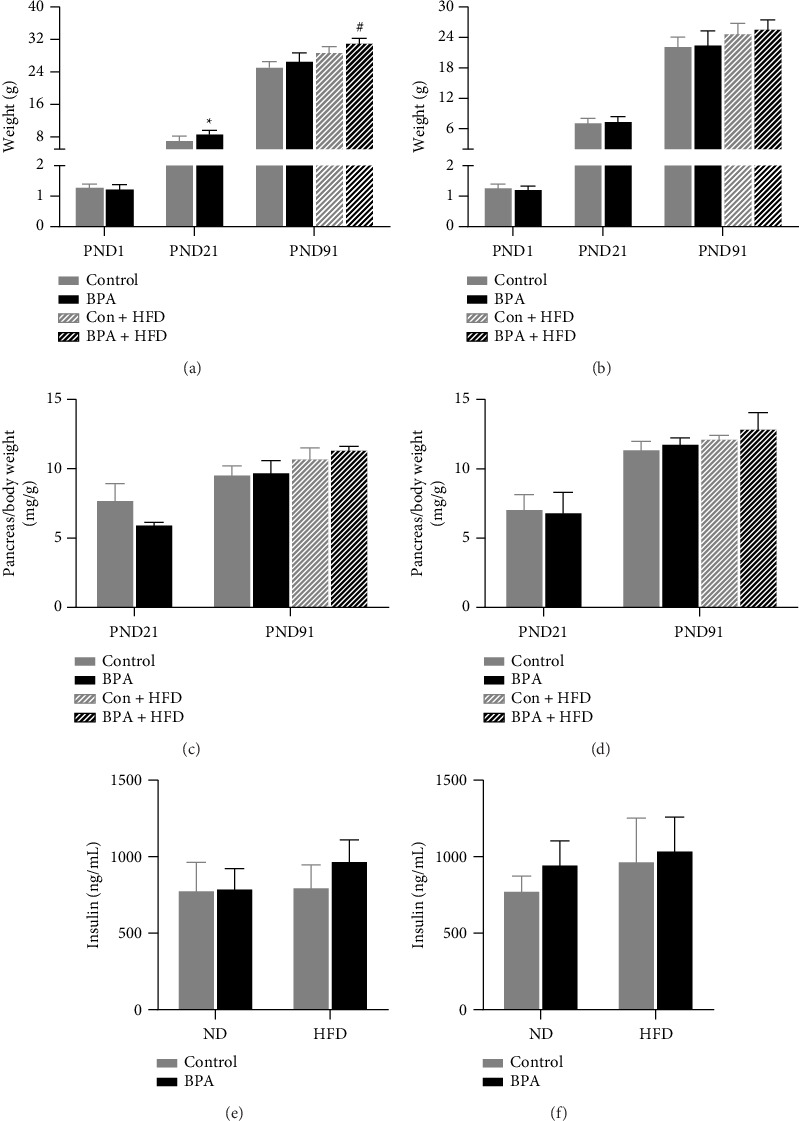
Body weight, pancreatic organ coefficient, and plasma insulin in mice. (a) Body weight of male mice. (b) Body weight of female mice. (c) Pancreatic organ coefficients of male mice. (d) Pancreatic organ coefficients of female mice. (e) Plasma insulin of male mice. (f) Plasma insulin of female mice. Data are shown as mean ± standard deviation and analyzed by two-way ANOVA, followed by Dunnett's post hoc pairwise comparison; ∗ indicates significant difference compared with Control, and # indicates significant difference compared with Con + HFD, *n* = 8.

Body weight, organ coefficients, and plasma insulin of the offspring mice are shown in Figure 1. In male mice, mice in the BPA group at PND21 had increased body weight compared with control (*p* < 0.05, Figure 1(a)); mice in the Con + BPA group at PND91 had increased body weight compared with Con + HFD (*p* < 0.05, Figure 1(a)). There was no statistically significant change in body weight in all groups of female mice (*p* > 0.05, Figure 1(b)). Pancreatic organ coefficient was decreased in the BPA group of male mice at PND21 (*p* < 0.05, Figure 1(c)). There was no statistically significant change in pancreatic organ coefficient in all groups of female mice (*p* > 0.05, Figure 1(d)). There was no statistically significant change in plasma insulin content in both male and female mice (*p* > 0.05, Figures 1(e) and 1(f)).

### 3.2. Early-Life BPA Exposure Promotes Disorders of Glucose Induced by Postweaning HFD in C57 BL/6J Mice

**Figure 2:**
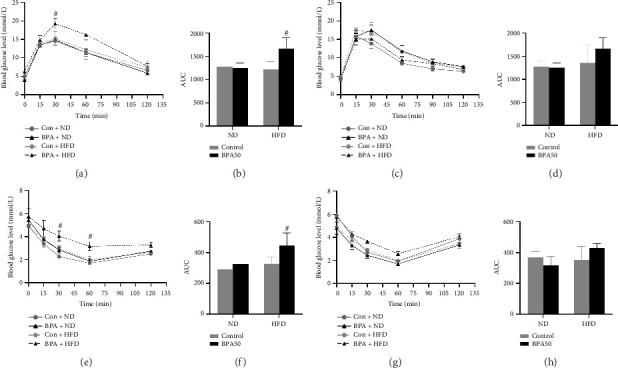
Early-life BPA exposure promotes impaired glucose tolerance and insulin tolerance in mice fed an HFD. (a) Changes in blood glucose levels of PND91 male mice in glucose tolerance test. (b) Area under the curve (AUC) of blood glucose change in glucose tolerance test of PND91 male mice. (c) Changes in blood glucose levels of PND91 female mice in glucose tolerance test. (d) Area under the curve (AUC) of blood glucose change in glucose tolerance test of PND91 female mice. (e) Changes in blood glucose levels of PND91 male mice in insulin tolerance test. (f) AUC of blood glucose change in insulin tolerance test of PND91 male mice. (g) Changes in blood glucose levels of PND91 female mice in insulin tolerance test. (h) AUC of blood glucose change in insulin tolerance test of PND91 female mice. Data are shown as mean ± standard deviation, and analyzed by two-way ANOVA, followed by Dunnett's post hoc pairwise comparison; ∗ indicates significant difference compared with Control, and # indicates significant difference compared with Con + HFD, *n* = 3.

To determine the effect of early-life BPA exposure in combination with postweaning HFD on glucose in mice, we conducted glucose and insulin tolerance tests at PND91 in mice. In the glucose tolerance test, male mice in the BPA + HFD group had significantly higher blood glucose levels than those in the Con + HFD group after 30 min of intraperitoneal glucose injection (*p* < 0.05, Figure 2(a)), and correspondingly, the area under the blood glucose curve was significantly higher in the BPA + HFD group than in the Con + HFD group (*p* < 0.05, Figure 2(b)); there was no statistically significant difference between the blood glucose at each time point and the area under the blood glucose curve in female mice (*p* > 0.05, Figures 2(c) and 2(d)). In the insulin tolerance test, male mice in the BPA + HFD group had significantly higher blood glucose levels than those in the Con + HFD group after 30 min and 60 min of intraperitoneal injection of insulin (*p* < 0.05, Figure 2(e)), and the area under the blood glucose curve was significantly higher than that in the Con + HFD group (*p* < 0.05, Figure 2(f)); there was no statistically significant difference between the blood glucose at each time point and the area under the blood glucose curve in female mice (*p* > 0.05, Figures 2(g) and 2(h)).

### 3.3. Early-Life BPA Exposure Promotes Pancreatic Islet Changes Induced by Postweaning HFD in C57 BL/6J Mice

**Figure 3:**
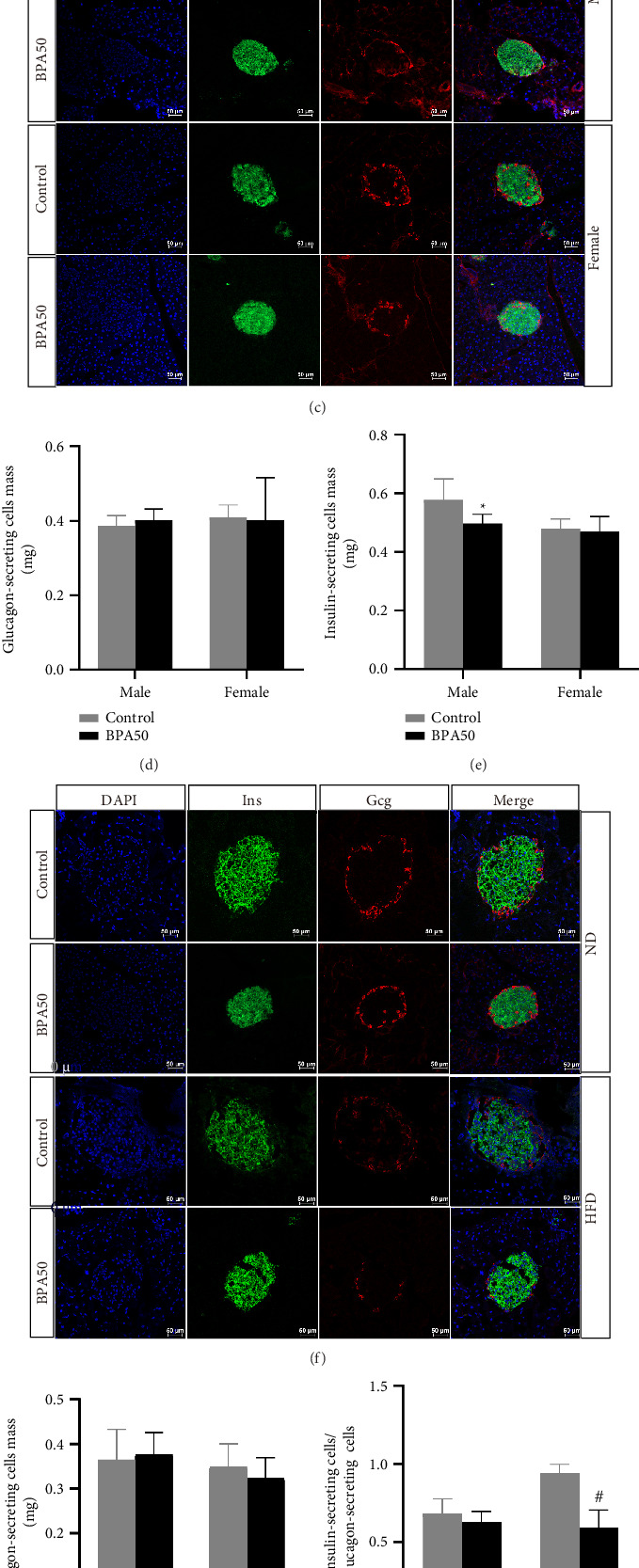
BPA promotes islet cell changes caused by HFD in early life. (a) Expression of pancreatic islet cell-related genes in male mice. (b) Expression of pancreatic islet cell–related genes in female mice. (c) Representative immunofluorescence images of insulin-secreting cells and glucagon-secreting cells in the islets of PND21 mice. (d) Mass of glucagon-secreting cells in the islets of PND21 mice. (e) Mass of insulin-secreting cells in the islets of PND21 mice. (f) Representative immunofluorescence images of insulin-secreting cells and glucagon-secreting cells in the islets of PND91 male mice. (g) Mass of glucagon-secreting cells in the islets of PND91 male mice. (h) Mass of insulin-secreting cells in the islets of PND91 male mice. (i) Representative immunofluorescence images of insulin-secreting cells and glucagon-secreting cells in the islets of PND91 female mice. (j) Mass of glucagon-secreting cells in the islets of PND91 female mice. (k) Mass of insulin-secreting cells in the islets of PND91 female mice. Data are shown as mean ± standard deviation, and analyzed by two-way ANOVA, followed by Dunnett's post hoc pairwise comparison; ∗ indicates significant difference compared with Control, and # indicates significant difference compared with Con + HFD, *n* = 5.

To elucidate the effects of early-life BPA exposure and postweaning HFD on pancreatic islets, we examined the expression levels of islet cell–related genes, as well as the mass of pancreatic insulin-secreting cells and glucagon-secreting cells. At PND21, *Ins1*, *Pdx1*, and *NeuroG3* gene expression was downregulated in male mice in the BPA group compared to the control group (*p* < 0.05, Figure 3(a)), while there was no statistical difference in the expression of each gene in female mice (*p* > 0.05, Figure 3(b)). mRNA expression of *Ins1*, *Pdx1*, and *NeuroG3* genes was downregulated in male mice in the BPA + HFD group compared to the Con + HFD group at PND91 (*p* < 0.05, Figure 3(a)), while there was no statistically significant difference in mRNA expression of each gene in female mice (*p* > 0.05, Figure 3(b)).

To clarify whether BPA exposure induces morphological damage to pancreatic islets in mice, we performed immunofluorescence staining of mouse pancreatic tissue sections and counted the mass of glucagon-secreting cells and insulin-secreting cells, and the results are shown in Figure 3. At PND21, compared with the control group, the mass of pancreatic insulin-secreting cells was decreased in the BPA group of male mice (*p* < 0.05, Figures 3(c) and 3(e)), and there was no statistically significant difference in the mass of pancreatic glucagon-secreting cells (*p* > 0.05, Figure 3(d)), whereas the mass of pancreatic insulin-secreting cells and insulin-secreting cells of female mice were not statistically different (*p* > 0.05, Figures 3(c)–3(e)). At PND91, the mass of insulin-secreting cells and glucagon-secreting cells was decreased in the BPA + HFD group of male mice compared to the Con + HFD group (*p* < 0.05, Figures 3(f)–3(h)), whereas the mass of insulin-secreting cells and glucagon-secreting cells was not significantly altered in female mice (*p* > 0.05, Figures 3(i)–3(k)).

### 3.4. Early-Life BPA Exposure Promotes Pancreatic Lipidomic Alterations Induced by Postweaning HFD in C57 BL/6J Mice

Lipid metabolism plays an important role in maintaining metabolic functions of the body. Therefore, we investigated whether early-life BPA exposure combined with postweaning HFD would cause abnormal lipid metabolism in the pancreas. Specifically, we performed lipidomics based on HPLC-MS/MS on pancreatic tissues of mice from different groups at PND21 and PND91. The fragmentation mode used in the lipidomics analysis was high-energy collision–induced dissociation (HCD), and the representative total ion chromatograms (TIC) are shown in Supporting Figure S1. The representative lipid matching process by mass spectrometry in the HPLC-MS/MS analysis is shown in Supporting Figure S2.

**Figure 4:**
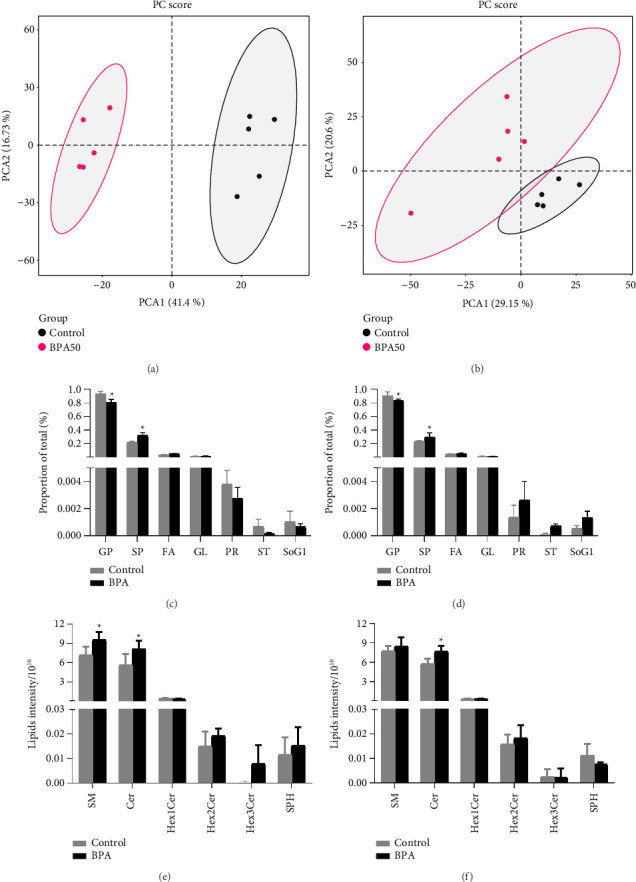
Early-life BPA exposure promotes pancreatic lipid metabolism changes. (a) Results of principal component analysis of pancreatic lipids in PND21 male mice. (b) Results of principal component analysis of pancreatic lipids in PND21 female mice. (c) Relative abundance of lipid classes in PND21 male mice. (d) Relative abundance of lipid classes in PND21 female mice. (e) Relative abundance of lipid subclasses in PND21 male mice. (f) Relative abundance of lipid subclasses in PND21 female mice. Shaded areas represent 95% confidence ellipses in (a) and (b). Data are shown as mean ± standard deviation, and analyzed by two-way ANOVA, followed by Dunnett's post hoc pairwise comparison; ∗ indicates significant difference compared with Control, and # indicates significant difference compared with Con + HFD, *n* = 5.

**Figure 5:**
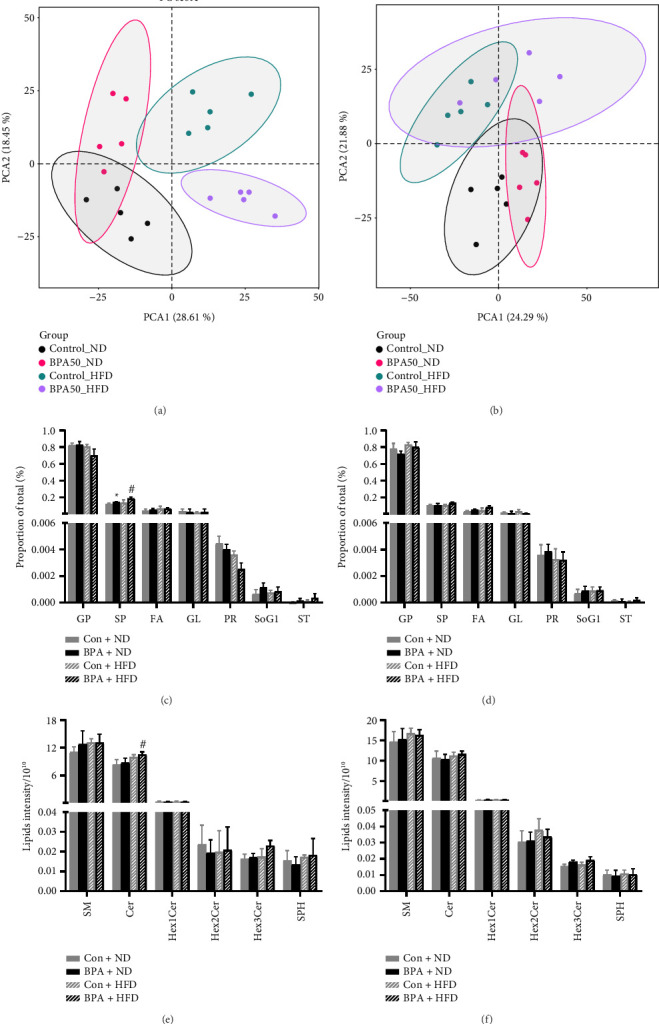
Early-life BPA exposure combined with postweaning high-fat diet promotes altered pancreatic lipid metabolism in mice. (a) Results of principal component analysis of pancreatic lipids in PND91 male mice. (b) Results of principal component analysis of pancreatic lipids in PND91 female mice. (c) Relative abundance of lipid classes in PND91 male mice. (d) Relative abundance of lipid classes in PND91 female mice. (e) Relative abundance of lipid subclasses in PND91 male mice. (f) Relative abundance of lipid subclasses in PND91 female mice. Shaded areas represent 95% confidence ellipses in (a) and (b). Data are shown as mean ± standard deviation, and analyzed by two-way ANOVA, followed by Dunnett's post hoc pairwise comparison; ∗ indicates significant difference compared with Control, and # indicates significant difference compared with Con + HFD, *n* = 5.

The score plot of PCA showed a clear distinction between BPA group and the control group (Figure 4(a)), which indicated that exposure to BPA causes severe disruption of lipid metabolism in PND21 male mice and failure to distinguish significantly between the control and BPA groups of female mice (Figure 4(b)). Classification analysis indicated that glycerophospholipids (GP) and sphingolipids (SP) are the most abundant identified lipid categories followed by fatty acyl (FA), glycerolipids (GL), prenol lipids (PR), sterol lipids (ST), and glucosylsphingosine (SoG1). In PND21 mice, the relative abundance of GP was decreased and the relative abundance of SP was increased in the male BPA group compared with the control group (*p* < 0.05, Figure 4(c)), whereas the difference in the relative abundance of lipids in female mice was not statistically significant (*p* > 0.05, Figure 4(d)). According to the lipid classification system proposed by the lipid metabolites and pathways strategy (LIPID MAPS) project, SP were classified as sphingomyelin (SM), Ceramides (Cer), hexosylceramide (HexCer), and sphingosine (SPH). In PND21 mice, the levels of SM and Cer in the male BPA-exposed group (Figure 4(e)), and Cer in the female BPA-exposed group (Figure 4(f)) were significantly higher than those in the control group (*p* < 0.05). At PND91, PCA showed that male mice could be better distinguished between groups, especially the Con + HFD group (Figure 5(a)); female mice could be distinguished in different dietary intervention groups, while the control and BPA groups failed to be significantly distinguished (Figure 5(b)). Lipid classification analysis showed that the SP abundance in the BPA + HFD group of male mice was significantly higher than that in the Con + HFD group (*p* < 0.05, Figure 5(c)), and the difference between the Con + HFD and BPA + HFD groups of female mice was not statistically significant (*p* > 0.05, Figure 5(d)). In the lipid subclasses, Cer abundance was significantly higher in the BPA + HFD group than in the Con + HFD group in male mice (*p* < 0.05, Figure 5(e)), while the difference between the Con + HFD group and the BPA + HFD group in female mice was not statistically significant (*p* > 0.05, Figure 5(f)).

### 3.5. Early-Life BPA Exposure Combined With Postweaning High-Fat Diet Increases Pancreatic Ceramide Ab Initio Synthesis

**Figure 6:**
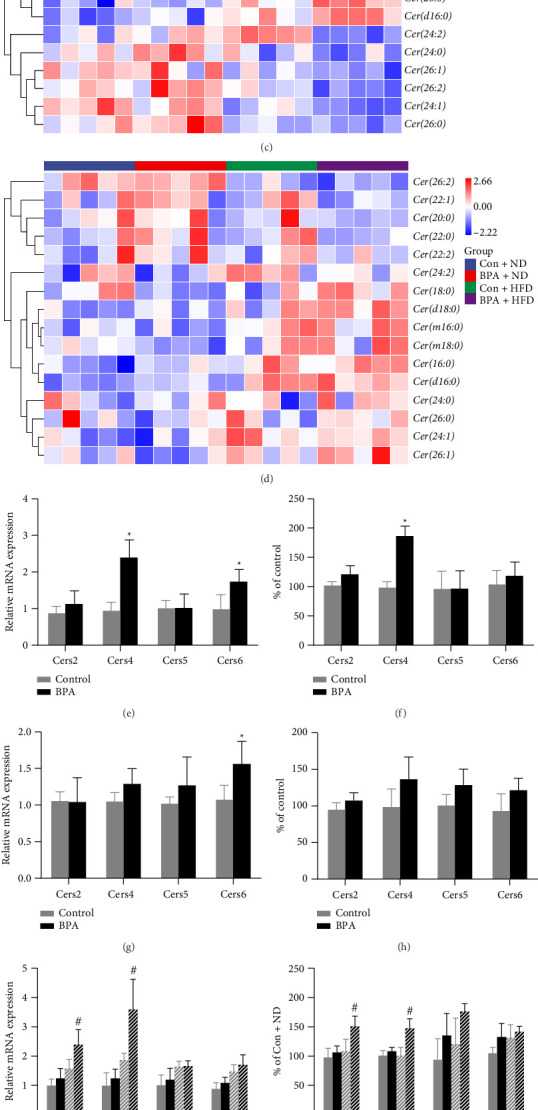
Early-life BPA exposure increases pancreatic ceramide de novo synthesis. (a) Abundance of ceramides with different acyl chain lengths in the pancreas of PND21 male mice. (b) Abundance of ceramides with different acyl chain lengths in the pancreas of PND21 female mice. (c) Abundance of ceramides with different acyl chain lengths in the pancreas of PND91 male mice. (d) Abundance of ceramides with different acyl chain lengths in the pancreas of PND91 female mice. (e) The mRNA expression level of ceramide synthase in the pancreas of PND21 male mice. (f) The activity of ceramide synthase in the pancreas of PND21 male mice. (g) The mRNA expression level of ceramide synthase in the pancreas of PND21 female mice. (h) The activity of ceramide synthase in the pancreas of PND21 female mice. (i) The mRNA expression level of ceramide synthase in the pancreas of PND91 male mice. (j) The activity of ceramide synthase in the pancreas of PND91 male mice. (k) The mRNA expression level of ceramide synthase in the pancreas of PND91 female mice. (l) The activity of ceramide synthase in the pancreas of PND91 female mice. Data are shown as mean ± standard deviation, and analyzed by two-way ANOVA, followed by Dunnett's post hoc pairwise comparison; ∗ indicates significant difference compared with Control, and # indicates significant difference compared with Con + HFD, *n* = 5.

The abundance of ceramide molecules with different acyl chain lengths was analyzed, and the results, as shown in Figure 6, showed an increase in the abundance of long-chain Cer (16 < C < 21) and a decrease in the abundance of very-long-chain Cer (C > 22) in both the BPA group of male mice with PND21(Figure 6(a)) and the BPA + HFD group of PND91 (Figure 6(c)), whereas a clear trend was observed for the female mice (Figures 6(b) and 6(d)).

Pancreatic ceramide synthase Cers2, Cers4, Cers5, Cers6 gene expression and enzyme activities were examined, and the results are shown in Figure 6. At PND21, mRNA expression of the *Cers4* gene was upregulated in the BPA group of male mice (*p* < 0.05, Figure 6(e)), and the enzyme activities of Cers4 were enhanced compared with those of the control group (*p* < 0.05, Figure 6(f)); There was no statistically significant difference in ceramide synthase gene mRNA expression enzyme activity in any of the PND21 female mice (*p* > 0.05, Figures 6(g) and 6(h)). mRNA expression of the *Cers2* and *Cers4* genes was upregulated in the male mouse BPA + HFD group at PND91 compared with the Con + HFD group (*p* < 0.05, Figure 6(i)), and Cers2 and Cers4 enzyme activities were enhanced (*p* < 0.05, Figure 6(j)); there was no statistical difference in mRNA expression of ceramide synthase gene and enzyme activity in the BPA + HFD group of PND91 female mice (*p* > 0.05, Figures 6(k) and 6(l)).

### 3.6. Pancreatic Ceramide Abundance Is Closely Related to Islet Cells Changes and Pancreatic Oxidative Damage in Male Mice

**Figure 7:**
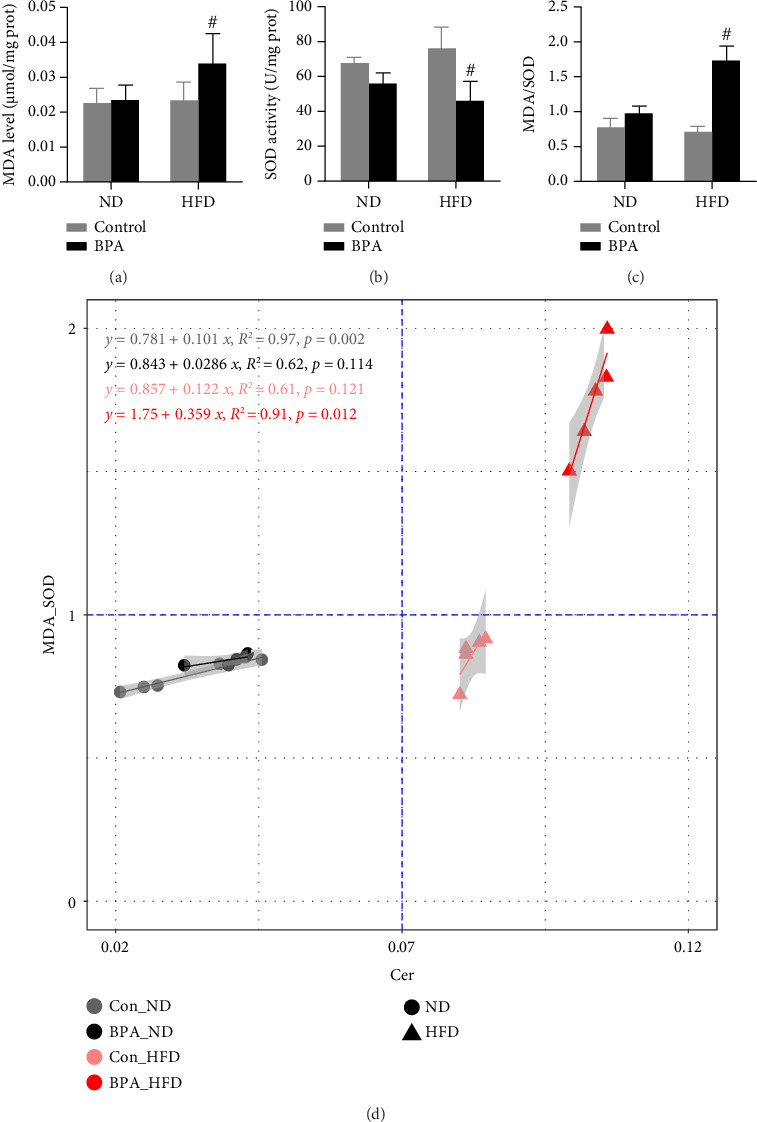
Increased pancreatic ceramide abundance in male mice is strongly associated with pancreatic oxidative damage. (a) MDA adduct in the pancreas of PND91 male mice. (b) SOD enzyme activity in the pancreas of PND91 male mice. (c) Ratio of MDA adduct and SOD enzyme activity in the pancreas of PND91 male mice. (d) Correlation analysis of ceramide abundance and the ratio of MDA adduct levels and SOD enzyme activity in the pancreas of PND91 male mice. Data are shown as mean ± standard deviation, and analyzed by two-way ANOVA, followed by Dunnett's post hoc pairwise comparison; ∗ indicates significant difference compared with Control, and # indicates significant difference compared with Con + HFD, *n* = 5.

Given that pancreatic ceramide abundance was significantly increased in male mice and the latter is closely associated with oxidative damage, we examined pancreatic MDA content and SOD enzyme activity in male mice. The results are shown in Figure 7. In PND91 male mice, MDA content was increased (*p* < 0.05, Figure 7(a)), SOD enzyme activity was decreased (*p* < 0.05, Figure 7(b)), and MDA to SOD ratio was increased (*p* < 0.05, Figure 7(c)) in the BPA + HFD group compared to the Con + HFD group. Finally, we did a correlation analysis between pancreatic MDA and SOD ratio and ceramide abundance in PND91 male mice, and the results showed a significant positive correlation between pancreatic MDA and SOD ratio and ceramide abundance (*p* < 0.05, Figure 7(d)).

## 4. Discussion

Extensive research has demonstrated that BPA can impact the metabolic regulation of lipids and carbohydrates. Owing to the robust metabolic capabilities of adults, exposure to environmental concentrations of BPA is unlikely to directly cause metabolic damage in this demographic. Conversely, pregnant women and their fetuses exhibit reduced activity of glucuronosyltransferase enzymes [14, 15], rendering them more susceptible to the effects of BPA exposure. Consequently, early-life exposure to BPA merits significant attention. The developmental phase in early life is a critical period for shaping the body's self-regulatory abilities, with metabolic regulation patterns established during this period persisting into adulthood and potentially throughout one's lifetime. In light of this, in the present study, BPA was administrated to mice (GD6-PND21) to observe the direct effects of early BPA exposure and its subsequent impact on lipid and carbohydrate metabolism under high-fat dietary stress after ceasing BPA exposure. This offers animal model evidence for the influence of adverse environmental factors during early life on the development of chronic diseases in adulthood.

BPA is characterized by its brief half-life within the organism, rapidly metabolizing into BPAG—a metabolite with negligible biological activity [25]. This study has identified the presence of bioactive, nonconjugated BPA in the placentas of mother mice and the livers of neonatal mice at PND1. This finding indicates that prenatal exposure to BPA at a dose of 50 μg/kg/d is sufficient for BPA to traverse the placental barrier and enter the fetal system. However, by PND21, nonconjugated BPA could no longer be detected in the F1 mice. This phenomenon could be attributed to the low efficiency of BPA transfer to the offspring through breast milk [26]. Additionally, following birth, both mother and offspring mice exhibit a marked increase in BPA metabolic capacity, leading to a substantial conversion of nonconjugated BPA into its conjugated form. Owing to the short half-life of BPA in the body, the levels of nonconjugated BPA in the offspring fell below the detection limit at PND91 following the cessation of exposure.

Morphological examination of pancreatic islets has shown that early-life exposure to BPA significantly alters the ratio of α-cells to β-cells, and this alteration persists over time. An HFD induces hyperglycemia and insulin resistance, which prompts compensatory proliferation of islet β-cells to meet insulin demands [27]. Notably, in PND91 male mice, this study observed a significant increase in insulin-secreting cells in the Con + HFD group compared to Con + ND, whereas the BPA + HFD group exhibited a decrease. These findings suggest that early-life BPA exposure impairs the compensatory hyperplasia of pancreatic β-cells in adult offspring mice in response to a high-fat diet, with a more pronounced effect in males. It has been found that BPA induces β-cell apoptosis [28]. ERαβ heterodimerization plays a key role in β-cell antiapoptosis, and the interaction of BPA with ERα and ERβ decreases ERαβ heterodimerization, leading to β-cell apoptosis [29]. Taken together, BPA may induce apoptosis in β-cells and increase the sensitivity of mice to a high-fat diet.

Findings from pancreatic lipidomics indicated that exposure to BPA early in life was strongly associated with altered pancreatic lipid profiles, most notably ceramide accumulation, and that these alterations persist. Of the numerous lipid accumulations induced by an HFD, SP such as ceramides are the most deleterious, regulating signaling and metabolic pathways that drive insulin resistance, triglyceride production, apoptosis, and fibrosis [30]. The effects of ceramides on the body's glycolipid metabolism manifest in several ways: first, ceramides inhibit the phosphorylation of insulin receptor substrate 1 (IRS-1) and block insulin signaling, leading to insulin insensitivity and insulin resistance in peripheral tissues (e.g., liver, muscle, and fat) [31]. Second, ceramide can induce apoptosis in pancreatic β-cells, reducing insulin secretion and synthesis, leading to pancreatic islet insufficiency [32]. Third, ceramide can promote de novo hepatic glucose and triglyceride synthesis, increasing blood glucose and lipid levels [33]. Fourth, ceramides can activate a chronic low-grade inflammatory response, increasing oxidative stress and endotoxin levels, further worsening metabolic disorders [34]. Several large population-based studies have established a correlation between blood ceramides and disorders of glucolipid metabolism [35–37]. In addition, ceramide levels in tissues such as liver, adipose, and skeletal muscle are also highly correlated with insulin resistance [33, 38]. Given the high correlation between ceramides and a variety of metabolic diseases, ceramides are now being used clinically as a biomarker for measuring a variety of metabolic diseases [39–41]. In addition, ceramides with different acyl chain lengths have distinct effects on body metabolism, and manipulation of ceramide acyl chain length by ceramide synthase can yield a wide range of functional and tissue-specific effects. According to available studies, Cer (16:0) and Cer (18:0) are reported to be most closely associated with a variety of metabolic diseases [42, 43]. As Bhagirath states, “Although many different sphingolipid species may contribute to the decline of tissue dysfunction in obesity and cardiometabolic diseases, ceramides containing both C16 and C18 acyl chains (C16:0 and C18:0) are particularly” [44].

To date, only a limited number of studies have focused on the relationship between BPA and ceramide metabolism. Among them, Wang et al. discovered in a population-based case–control study that a specific isoform of ceramide (C16-cer) mediated the association between BPA and obesity, obesity-associated insulin resistance, and adipose tissue inflammation; further animal experiments demonstrated that 8-week exposure to BPA (1000 nM) promoted ceramide accumulation in adipose tissue, activated PKCζ, promoted adipose tissue inflammation, increased proinflammatory cytokine expression and secretion via the JNK/NF-κB pathway, and reduced insulin sensitivity by disrupting IRS1-PI3K-AKT signaling in HFD-fed mice, whereas myricetin (an inhibitor of ceramide synthase) was found to inhibit BPA-induced adipose tissue inflammation and insulin resistance [45]. This study noted that BPA exposure induced enhanced ceramide ab initio synthesis and exacerbated metabolic disturbances during obesity; however, it did not explore alterations in pancreatic islets during this period. Many previous studies have established that ceramide plays an important role in pancreatic β-cell apoptosis [43, 46]. In this study, we also observed a decrease in β-cell mass in PND21 mice. In summary, BPA may also induce β-cell apoptosis by interfering with neural metabolism.

An HFD results in disturbances in lipid metabolism in the body, elevating free radical production and lipid peroxidation, thus causing oxidative stress [47]. Oxidative stress is characterized by the excessive production of reactive oxygen radicals (ROS) and reactive nitrogen radicals (RNS) in the body, which exceeds the scavenging capacity of the antioxidant system, resulting in cell and tissue damage [48, 49]. An HFD induces and exacerbates oxidative stress through multiple pathways. On the one hand, it elevates plasma free fatty acid (FFA) levels, and FFA can enter mitochondria to participate in β-oxidation, leading to the overproduction of ROS in mitochondria [50]. On the other hand, it prompts immune cells such as macrophages and neutrophils to release pro-inflammatory cytokines, e.g., TNF-α and IL-6, which can activate enzyme systems such as NADPH oxidase and xanthine oxidase and augment the production of ROS and RNS [51]. Additionally, an HFD impacts the structure and function of the intestinal microbial community, leading to an elevation in intestinal permeability and the entry of lipopolysaccharide (LPS) into the circulation, where it binds to TLR4 and triggers inflammatory responses and oxidative stress [52]. In view of this, the present study assessed MDA levels, SOD enzyme activity, and total antioxidant capacity in the pancreas of PND91 mice and measured the pancreatic oxidative and antioxidant balance by the ratio of the two. The results demonstrated that early-life BPA exposure exacerbated lipid peroxidation oxidative damage in the pancreas of mice fed an HFD and caused an imbalance between oxidative and antioxidant levels. Finally, pancreatic ceramide abundance was found to be strongly associated with islet structural alterations as well as pancreatic oxidative/antioxidant levels by correlation analysis.

Notably, most observed effects in this study were statistically significant exclusively in male mice, with female counterparts exhibiting no marked alterations. This finding aligns with the documented gender-specific biological responses to BPA exposure reported in previous investigations. Marta et al. demonstrated that subcutaneous administration of BPA (10 μg/kg/day, GD9–GD16) induced glucolipid metabolic disorders in adult male offspring, characterized by increased body weight, elevated fasting glucose, impaired glucose tolerance, elevated plasma FFAs, and altered gene expression in white adipose tissue and liver. When these BPA-exposed male offspring were subsequently challenged with a high-fat diet, they exhibited exacerbated pancreatic β-cell dysfunction. In contrast, female offspring showed no significant changes in glucose metabolic homeostasis under identical experimental conditions [53]. Another investigation employing oral BPA administration (100 μg/kg/day) from GD6 to PND21, followed by high-fat feeding postweaning, revealed that BPA induced hepatic lipid metabolic dysfunction in male offspring at birth through sex-specific epigenetic mechanisms involving DNA methylation and histone modifications of the hepatic *Cpt1a* gene. This dysfunction progressively worsened with adult high-fat diet exposure, culminating in aggravated diet-induced fatty liver pathology. Again, female offspring demonstrated no comparable susceptibility [54].

The sexually dimorphic effects of BPA may involve two primary mechanisms. First, sex-specific differences in BPA pharmacokinetics likely contribute significantly. Existing evidence indicates higher serum BPA concentrations in males compared to females [55]. This disparity may arise from androgen-mediated inhibition of UGT enzymes, which are critical for BPA detoxification and clearance [56]. Second, BPA's structural similarity to endogenous estrogens enables it to modulate estrogen receptor signaling pathways, thereby perturbing metabolic homeostasis [57]. Males may exhibit heightened metabolic sensitivity to BPA due to the absence of the protective buffering conferred by elevated endogenous estrogen levels in females.

## 5. Conclusion

Early-life exposure to BPA may increase sensitivity to a high-fat diet in offspring. The mechanism may be closely related to the combined exposure to BPA and high-fat diet, which alters the expression of pancreatic functional genes and the morphology of pancreatic islets, interferes with pancreatic ceramide metabolism, and increases susceptibility to high-fat diet, with significant sex differences. Our findings suggest that controlling exposure to BPA early in life is an effective strategy to protect metabolic capacity in people on high-fat diets, and that neurosphingolipid synthase may be a potential target to mitigate the adverse effects of BPA exposure.

## Figures and Tables

**Table 1:** Grouping of offspring mice.

	BPA	Diet
Mice	C57BL/6J	

Time	GD6-PND21	PND22-PND91

Treatment	0, 50 μg/kg/day BPA	ND (D12450J)
		HFD (D12492)

Group (half male and half female)	Control, BPA (*n* = 10)	Con + ND, BPA + ND, Con + HFD, BPA + HFD (*n* = 10)

**Table 2:** Multiple reaction monitoring (MRM) parameters.

Target compound	Parent ion (*m*/*z*)	Product ion (*m*/*z*)	Collision energy (eV)	Cone voltage (V)
BPA	227.1	212	18	112
		133.1	26	112

13C12-BPA	229.2	213	18	107
		135	26	107

**Table 3:** The concentrations of free BPA.

Time	Sample	Control (ng/mL)	BPA (ng/mL)
PND1	F0-plasma	ND	18.52 ± 6.94
	F0-liver	ND	33.01 ± 17.56
	Placenta	ND	17.36 ± 2.16
	F1-liver	ND	4.14 ± 2.49

*Note: n* = 3.

Abbreviation: ND = no detection.

## Data Availability

The data that support the findings of this study are available upon request from the corresponding author. The data are not publicly available due to privacy or ethical restrictions.

## Supporting Information

The Supporting Information, including Tables S1–S3 and Figures S1 and S2, is available online in the Supporting Information section.
