# Supplementary material for: Early-Life Exposure to Bisphenol A Damaged Pancreas That May Increase Offspring Sensitivity to High-Fat Diets
Source: J Toxicol. 2025 Aug 13;2025:6189790. doi: 10.1155/jt/6189790 (PMC12367390; doi:10.1155/jt/6189790)
Supplement: Supporting Information — Additional supporting information can be found online in the Supporting Information section. [file 6189790.f1.docx]

Table S1. Pregnancy and delivery outcomes of the dams

| Time | Group | Vaginal plug (+) | Live  Birth  (Litters) | Stillbirth  (Litters) | Inclued  analysis | Number of offsping mice/Litters | |
| --- | --- | --- | --- | --- | --- | --- | --- |
|  |  |  |  |  |  | Number of male offsping mice/Litters | Number of female offsping mice/Litters |
| GD6-PND21 | Control | 10 | 9 | 1 | 10 | 2 (0, 3) | 2 (1, 4) |
|  | BPA | 10 | 8 | 3 | 10 | 3 (1, 3) | 2 (1, 4) |
| PND21-PND91 | Control | 22 | 20 | 2 | 16 | 2 (1, 4) | 2 (1, 5) |
|  | BPA | 22 | 20 | 0 | 16 | 2 (2, 4) | 2 (1, 4) |

The determination time of stillbirth was that after the delivery of pregnant mice, the mice were wrapped by the placenta, cyanosis, no heartbeat breathing, and the occurrence of stillbirth nest was excluded from the experiment. Stillbirth nest not all pups died, eating pups behavior occurred in the stillbirth nest, eating pups nest has been excluded with the stillbirth nest experiment.

Table S2. Food and water intake of the dams

| Time | Group | Number of animals | Water intake (mL) | Food intake (g) |
| --- | --- | --- | --- | --- |
| GD6-PND21 | Control | 8 | 4.49 ± 0.57 | 4.16 ± 0.70 |
|  | BPA50 | 5 | 4.00 ± 0.50 | 4.07 ± 0.35 |
| PND21-PND91 | Control | 16 | 4.62 ± 0.42 | 4.83 ± 0.41 |
|  | BPA50 | 16 | 4.37 ± 0.29 | 4.89 ± 0.32 |

Table S3. Sequences of primers used in quantitative RT-PCR

| Target gene | Primer | Nucleotide sequence |
| --- | --- | --- |
| *Cers2* | F | 5’-ATGCTCCAGACCTTGTATGACT-3’ |
|  | R | 5’-CTGAGGCTTTGGCATAGACAC-3’ |
| *Cers4* | F | 5’-TACCCACATCAGACCCTGAAT-3’ |
|  | R | 5’-TGAAGTCCTTGCGTTTGACATC-3’ |
| *Cers5* | F | 5’-CGGGGAAAGGTGTCTAAGGAT-3’ |
|  | R | 5’-GTTCATGCAGTTGGCACCATT-3’ |
| *Cers6* | F | 5’-GATTCATAGCCAAACCATGTGCC-3’ |
|  | R | 5’-AATGCTCCGAACATCCCAGTC-3’ |
| *Dnmt1* | F | 5’- AAGAATGGTGTTGTCTACCGAC-3’ |
|  | R | 5’- CATCCAGGTTGCTCCCCTTG-3’ |
| *Gcg* | F | 5’-CTCACAGGGCACATTCACCA-3’ |
|  | R | 5’-TGGCAATGTTGTTCCGGTTC-3’ |
| *Ghrl* | F | 5’- GACAAGTAACCACGGACAG-3’ |
|  | R | 5’- TTGGCATACAGGTGAACAT-3’ |
| *Ins1* | F | 5’-CACTTCCTACCCCTGCTGG-3’ |
|  | R | 5’-ACCACAAAGATGCTGTTTGACA-3’ |
| *Neurog3* | F | 5’-CCAAGAGCGAGTTGGCACT-3’ |
|  | R | 5’-CGGGCCATAGAAGCTGTGG-3’ |
| *Pdx1* | F | 5’-CCCCAGTTTACAAGCTCGCT-3’ |
|  | R | 5’-CTCGGTTCCATTCGGGAAAGG-3’ |
| *Ppy* | F | 5’- TCTCCCTGTTTCTCGTATCCAC-3’ |
|  | R | 5’- GCATAGTCGCCTGGGTACA-3’ |
| *Sst* | F | 5’- GTCTGCCAACTCGAACCCA-3’ |
|  | R | 5’- AGATATGGGGTTTGGGGGAGA-3’ |
| *β-actin* | F | 5’-GATTACTGCTCTGGCTCCTAGC-3’ |
|  | R | 5’-GACTCATCGTACTCCTGCTTGC-3’ |


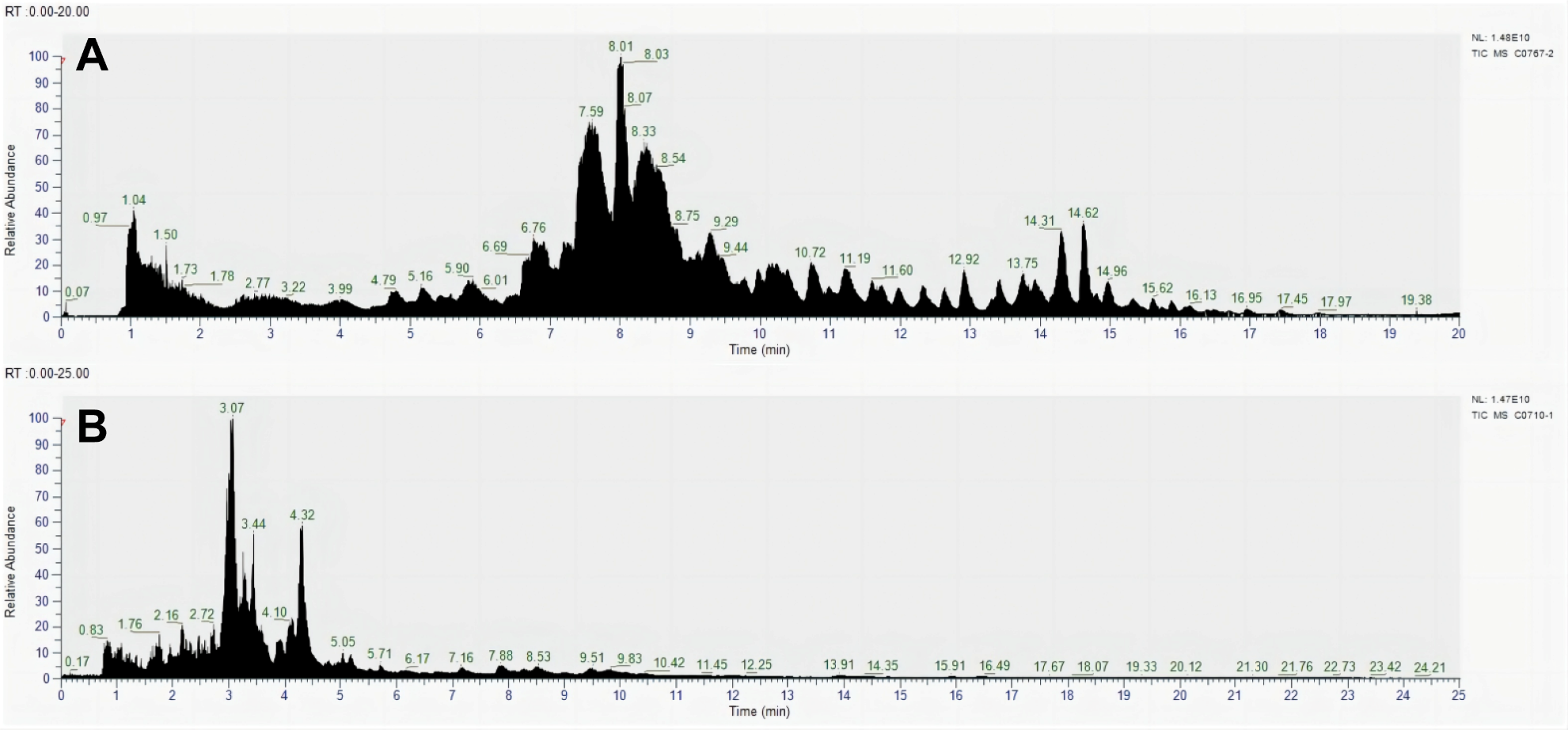


Figure S1 Representative total ion chromatograms in positive ion (A) and negative ion (B) modes, with the horizontal axis indicating retention time and the vertical axis indicating the relative abundance of various lipids.


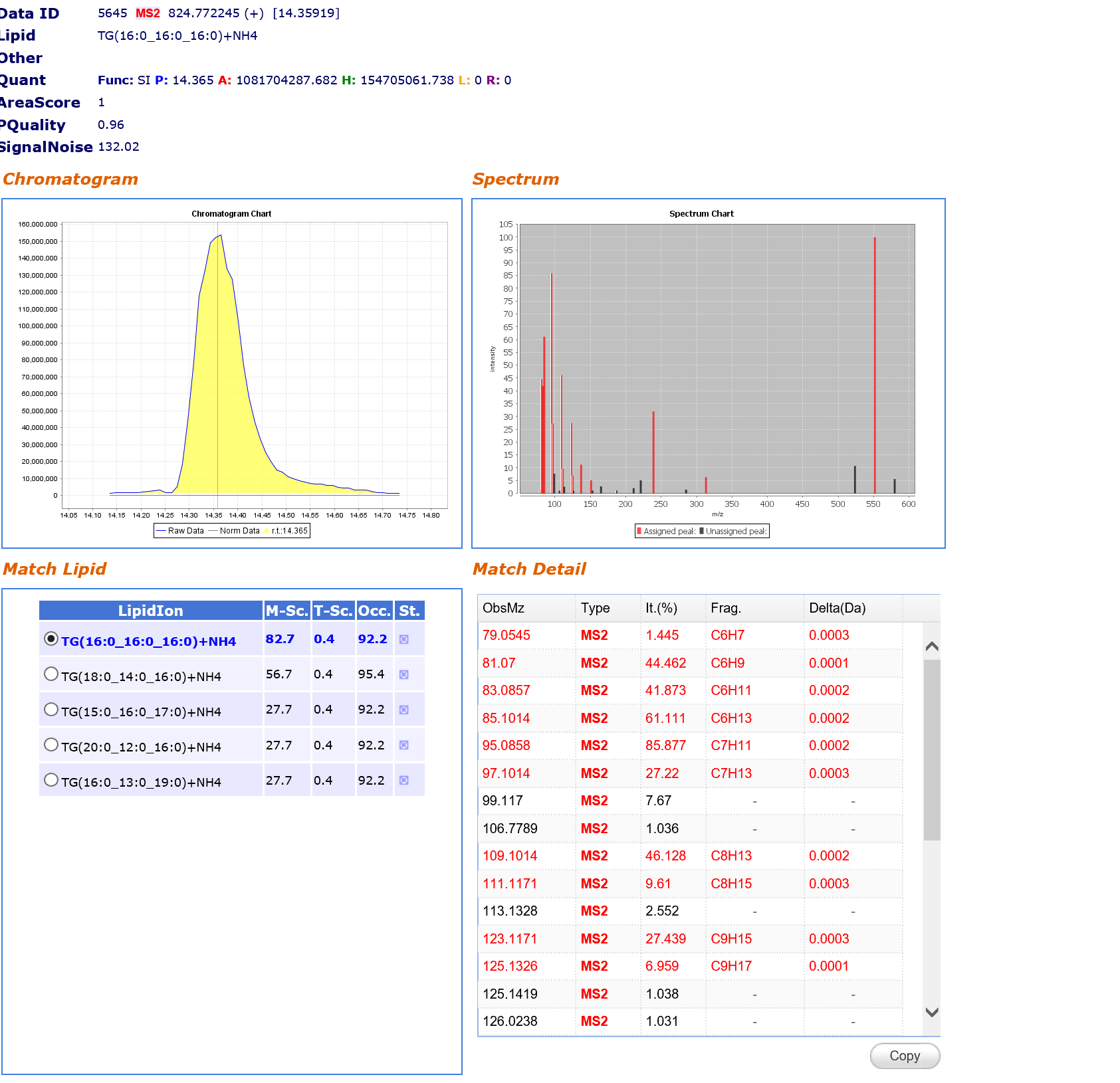


Figure S2 Representative pancreatic lipid matching process.


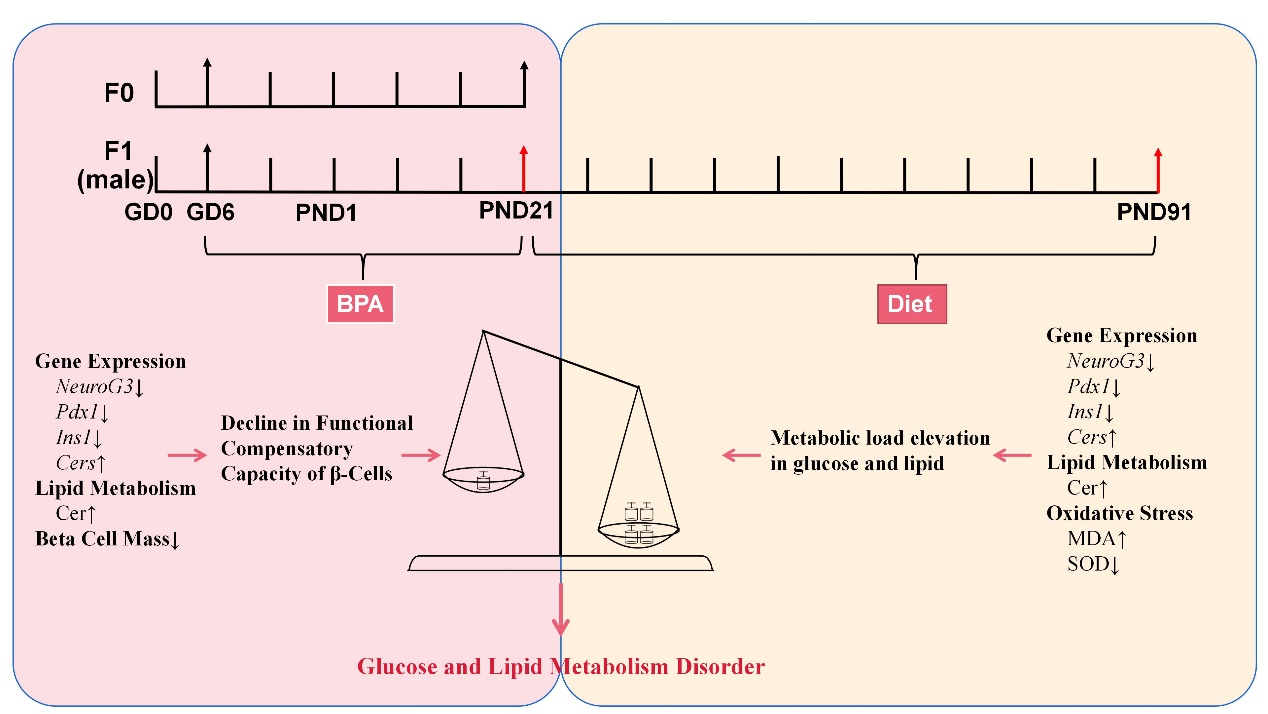


Graphical abstract. Pregnant mice were exposed to 50 μg/kg/day of BPA via drinking water from GD6 to PND21. After weaning, BPA exposure was terminated, and a HFD intervention was initiated. At PND21, offspring mice exhibited altered pancreatic gene expression, ceramide accumulation, and reduced pancreatic β-cell mass. These alterations impaired the functional compensation of pancreatic β-cells, rendering them unable to adapt to the metabolic stress induced by HFD, ultimately leading to dysregulation of glucose and lipid metabolism in adulthood (PND91). Importantly, most of these changes were observed exclusively in male mice, with no significant abnormalities detected in female mice.
